# C/N ratio and carbon source-dependent lipid production profiling in *Rhodotorula toruloides*

**DOI:** 10.1007/s00253-020-10386-5

**Published:** 2020-01-24

**Authors:** Helberth Júnnior Santos Lopes, Nemailla Bonturi, Eduard Johannes Kerkhoven, Everson Alves Miranda, Petri-Jaan Lahtvee

**Affiliations:** 1 Institute of Technology, University of Tartu, Tartu, Estonia; 2 Department of Materials and Bioprocess Engineering, School of Chemical Engineering, State University of Campinas, Cidade Universitária Zeferino Vaz - Barão Geraldo, Campinas, SP 13083-970 Brazil; 3 Department of Biology and Biological Engineering, Chalmers University of Technology, Göteborg, Sweden; 4 Novo Nordisk Foundation Center for Biosustainability, Chalmers University of Technology, Göteborg, Sweden

**Keywords:** *Rhodotorula toruloides*, Microbial oil, Biorefineries, Genome-scale metabolic model, Flux balance analysis, Carbon to nitrogen ratio, Alternative substrates

## Abstract

Microbial oils are lipids produced by oleaginous microorganisms, which can be used as a potential feedstock for oleochemical production. The oleaginous yeast *Rhodotorula toruloides* can co-produce microbial oils and high-value compounds from low-cost substrates, such as xylose and acetic acid (from hemicellulosic hydrolysates) and raw glycerol (a byproduct of biodiesel production). One step towards economic viability is identifying the best conditions for lipid production, primarily the most suitable carbon-to-nitrogen ratio (C/N). Here, we aimed to identify the best conditions and cultivation mode for lipid production by *R. toruloides* using various low-cost substrates and a range of C/N ratios (60, 80, 100, and 120). Turbidostat mode was used to achieve a steady state at the maximal specific growth rate and to avoid continuously changing environmental conditions (i.e., C/N ratio) that inherently occur in batch mode. Regardless of the carbon source, higher C/N ratios increased lipid yields (up to 60% on xylose at a C/N of 120) but decreased the specific growth rate. Growth on glycerol resulted in the highest specific growth and lipid production (0.085 g lipids/gDW*h) rates at C/Ns between 60 and 100. We went on to study lipid production using glycerol in both batch and fed-batch modes, which resulted in lower specific lipid production rates compared with turbisdostat, however, fed batch is superior in terms of biomass production and lipid titers. By combining the data we obtained in these experiments with a genome-scale metabolic model of *R. toruloides*, we identified targets for improvements in lipid production that could be carried out either by metabolic engineering or process optimization.

**Electronic supplementary material:**

The online version of this article (10.1007/s00253-020-10386-5) contains supplementary material, which is available to authorized users.

## Introduction

The transition towards a bioeconomy requires novel processes for chemical, material, and liquid fuel production that use sustainable substrates, have improved life cycle assessments, and require less energy to produce. Recent developments have drawn attention to the production of oleochemicals, i.e., chemicals derived from plant oils and animal fats, as attractive alternative feedstocks in the petrochemical industry (Adrio 2017; Unrean et al. 2017; Yang et al. 2018). Oleochemicals comprise a wide range of molecules that can be used as biofuels (fatty acid methyl esters, i.e., biodiesel), cosmetics, plastics, surface coatings, surfactants, lubricants, and paints, among others. The global demand for fatty acids and fatty alcohols in the year 2020 is expected to reach over 10 Mt, and the projected growth of global biodiesel production is also driving the need to increase the production of fatty acid methyl esters (Yang et al. 2018). Microbial lipids are one fatty acid source that is also considered to be a potential feedstock for oleochemical production (Unrean et al. 2017). Microbial oils, also termed single cell oils (SCOs), are mostly composed of triacylglycerides (TAGs), and are produced by oleaginous bacteria, algae, yeasts, and molds which are capable of accumulating more than 20% of their dry biomass as lipids (Ratledge and Wynn 2002). Production of SCOs does not require land or other resources used for food production and is not affected by climate or season (Koutinas et al. 2014). Lipid accumulation occurs when oleaginous microorganisms are cultivated in a medium with an excess of carbon where other nutrients, particularly nitrogen, is limiting their growth. Therefore, the carbon-to-nitrogen ratio (C/N) plays an important role in triggering lipid accumulation (Beopoulos et al. 2011; Papanikolaou and Aggelis 2011).

Among the oleaginous microorganisms, the yeast *Rhodotorula toruloides* (formerly known as *Rhodosporidium toruloides*) has recently been considered a workhorse for biotechnological applications (Park et al. 2017). This yeast can accumulate up to 70% of its dry biomass in lipids and can be grown to high cell densities (Li et al. 2007). *R. toruloides* is also a natural producer of industrially relevant and high-value compounds such as carotenoids, l-phenylalanine ammonia-lyase, and d-amino acid oxidase (Park et al. 2017). This strain is also capable of metabolizing various substrates (Papanikolaou and Aggelis 2011), including non-detoxified lignocellulosic hydrolysates (Bonturi et al. 2017). The ability to produce lipids and high-value co-products from a low-cost substrate is an important economic consideration when producing biodiesel from microbial oils (Koutinas et al. 2014). Examples of potential low-cost substrates for microbial oil production are hemicellulosic hydrolysates and raw glycerol. Hemicellulose is the second most abundant fraction of lignocellulose and its extraction and hydrolysis produces mainly xylose (along with other C5 and some C6 sugars) and acetic acid (Chandel et al. 2013). Raw glycerol is the main byproduct of biodiesel production (approximately 10%, *w*/*w*; Papanikolaou et al. 2008). Their use can be integrated into biorefineries and, hence, promote the circular economy.

Because the lipid content of cells is influenced by several factors, such as the strain, carbon source, C/N ratio, medium components, and cultivation conditions (Papanikolaou and Aggelis 2011), it is important to identify the best conditions for lipid production. Most preliminary studies employ batch cultivation in flasks to determine the optimal conditions, including the C/N ratio. During batch cultivation, microorganisms constantly switch their physiological status in response to changes in external conditions (Shen et al. 2013), such as depletion of nutrients, accumulation of toxic compounds, and changes in the ionic balance due to changes in pH, any one of which might result in growth limiting conditions (Monod 1949). For example, one characterization of the physiology of *R. toruloides* CCT 0783 cultivated in batch using glycerol showed that an increase in extracellular pH (from 6.0 to 7.3) during cultivation had a limiting effect on the growth of this yeast before the exhaustion of the carbon source (Azambuja et al. 2018). Careful characterization of the physiology of lipid accumulation is also crucial for scaling up microbial oil and oleochemical production. To acquire more precise data, continuous cultivations is often a more suitable strategy for this purpose because it offers better control of the variables that define the growth environment (Shen et al. 2017).

Only a small number of studies have used continuous cultivation to characterize oleaginous yeasts (Anschau et al. 2014; Béligon et al. 2016; Kerkhoven et al. 2016), and very few have focused on *R. toruloides* (Shen et al. 2013; Shen et al. 2017). The objective of this work was to identify the best conditions for lipid production by the yeast *R. toruloides* using various carbon sources and a continuous cultivation mode which is suitable for physiology studies. The low-cost substrates xylose, acetic acid, and glycerol were chosen as the sole carbon sources (i.e., hemicellulosic hydrolysates and raw glycerol). A further aim was to use the cultivation data together with genome-scale metabolic model of *R. toruloides* to gain the first holistic insight into the intracellular consumption patterns used to metabolize these three substrates.

## Materials and methods

### Strain, inoculum, and materials

*Rhodotorula toruloides* CCT 0783 (Coleção de Culturas Tropicais, Fundação André Tosello, Campinas, Brazil; synonym IFO10076) was used throughout the study. Aliquots of 0.5 mL stock culture, mixed with 10% glycerol (*w*/*v*) were stored at − 80 °C. The inoculum was prepared in batch cultivation on YPD in triplicate flasks at 200 rpm and 30 °C. Cells were washed twice with NaCl 0.9% (wt/vol) prior to inoculation. Turbidostat and fed-batch cultivation were carried out in MiniBio 1000 1.0 L bioreactors (Applikon Biotechnology, Delft, The Netherlands) equipped with temperature control, dissolved oxygen sensor, BugLab BE3000 Biomass Monitor (Bug Lab, Concord, CA, USA), and CO_2_ and O_2_ off-gas monitor (BlueInOne, BlueSens, Herten, Germany). Data was collected and processed with BioXpert V2 software v. 2.95 (Applikon Biotechnology, Delft, The Netherlands).

### Turbidostat cultivations

Turbidostat cultivations were carried out using basal mineral medium, consisting of (g/L): KH_2_PO_4_, 3.0; MgSO_4_·7H_2_O, 0.5; 1 mL of trace elements; and 1 mL of vitamin solution (Lahtvee et al. 2017). The basal medium was supplemented with one nitrogen (ammonium sulfate) and one carbon (acetic acid, glycerol or xylose) source. For the initial batch phase, 10 g of carbon and 5 g of nitrogen source/L were added to the basal medium, resulting in a C/N ratio of 4.5 (mol/mol) and inoculated with 10% of culture volume. For turbidostat phase, the amount of nitrogen was adjusted in the medium to reach the desired C/N ratio. Fresh medium inflow in turbidostat was started after the culture had reached a desired OD value in batch. Samples were collected under steady-state conditions from a single experiment where on-line parameters (CO_2_, O_2_, OD) had been constant for at least three residual volumes. The pH of the culture was maintained at 6.0 by adding 2 mol/L KOH solution and temperature was kept at 30 °C. All cultivations were kept aerobic, by maintaining measured pO_2_ levels above 30%.

### Batch cultivation using glycerol as a carbon source

Cultivations were performed in shake flasks for 96 h, and samples were withdrawn every 24 h. The medium was composed of the following (g/L): glycerol, 70.0 or 10.0; MgSO_4_·7H_2_O, 1.5; KH_2_PO_4_, 1.5; yeast extract (containing 3.8% of ammoniacal nitrogen and 10.5% of total nitrogen), 2.0; and (NH_4_)_2_SO_4_, 0.4. Trace metal solution (Meesters et al. 1996) was added in the medium at a final concentration of 1.0% (vol/vol). The C/N molar ratio of the medium was 100, and the initial pH was adjusted to 6.0 using 2 mol/L NaOH solution.

### Fed-batch cultivations using glycerol as carbon source

The fed-batch cultivation began with a batch phase using the same basal mineral medium as described before, except for (NH_4_)_2_SO_4,_ which was added to achieve the desired initial C/N ratio. Glycerol at 40 g/L was used as a sole carbon source. Experiments were carried out in duplicate. Concentrated mineral medium with the C/N ratio of 60 (mol/mol) supplemented with glycerol was used for feeding at the flow rate profile according to the Eq. (1):

1 $$ F(t)=\frac{Y_{XS}{\mu}_0}{c_f^s-{c}_s}{x}_0{V}_0{e}^{\mu_0t} $$

where *F*(*t*) is the flow rate (mL/h), *Y*_XS_ is the glycerol consumed per biomass (g glycerol/gDW), *μ*_0_ is the specific growth rate (1/h), *c*_f_^s^ and *c*_s_ are the glycerol concentration in the feeding medium and vessel (g/L), respectively, *x*_0_ is the biomass concentration (gDW/L) at the end of batch phase, *V*_0_ is the volume at the beginning of the fed batch (L), and *t* is time (h). Three different experimental set-ups were used: (1) molar C/N ratio of 60 during batch and glycerol feeding concentration of 190 g/L and *μ*_0_ of 0.03 or (2) 0.05 1/h and (3) molar C/N ratio of 17 during batch and glycerol feeding concentration of 220 g/L and *μ*_0_ of 0.05 1/h. The initial working volume was 0.5 L, pH was kept at 5.5 by the addition of 2 M KOH, dissolved oxygen was controlled to be over 35%, and temperature setpoint was 30 °C.

### Analytical methods

Cell growth in bioreactors was monitored by immersible optical biomass sensor at 1300 nm, and the dry cellular weight was measured gravimetrically. Concentrations of xylose, xylitol, pyruvate, acetic acid, and glycerol were quantified by HPLC (Prominex, Shimadzu, Kyoto, Japan) equipped with a refractive index detector RID-10A (Shimadzu, Kyoto, Japan) using an HPX-87H (Biorad, Hercules, USA) column at 45 °C, 5 mM H_2_SO_4_ at 0.6 mL/min as the mobile phase, and 10 μL injection volume.

Lipids were extracted by the methodology based on Folch et al. (1953) as described in Bonturi et al. (2015) and quantified gravimetrically. A rotary evaporator R-200 (Buchi, Switzerland) was used for solvent evaporation for the quantification method. One milliliter of culture broth was centrifuged at 1500×*g* for 15 min at 4 °C. The supernatant was diluted and used for extracellular protein quantification. For intracellular protein measurement, the cell mass was resuspended in 0.25 mL of Y-PER (Pierce, Rockford, USA) and incubated at 27 °C for 20 min, followed by lysis with glass beads (425–600 μm diameter, Sigma-Aldrich, St. Louis, USA) using Fastprep system (FastPrep-24, M.P Biomedicals, Irvine, California, USA) in three rotations of 20 s at maximal speed. The debris were separated after lysis by centrifugation at 3000×*g* for 5 min at 4 °C. Intra- and extracellular cell proteins were quantified using BCA and Bradford assays (Pierce, Rockford, USA) using a BSA standard calibration curve (curve ranged from 0 to 2000 μg/mL).

Carotenoids were extracted from *R. toruloides* lyophilized cells according to Lee et al. (2014). The quantification was carried out by HPLC Dionex Ultimate 3000 model (ThermoFisher, Massachusetts, USA) with an online diode-array detector DAD 3000, acclaim 120 C18 column (ThermoFisher, Massachusetts, USA) at 40 °C and a mix of acetone and water as mobile phase with proportions of acetone to water 80:20 (vol/vol) from 0 to 5 min (ascending slope), 100:0 from 5 to 10 min (plateau), and 80:20 from 10 to 11 min (descending slope; Weber et al. 2007).

### Genome-scale modeling

Internal metabolic flux distributions were estimated with the *R. toruloides* metabolic network at genome scale (Tiukova et al. 2019): version 1.2.1. of rhto-GEM (https://github.com/SysBioChalmers/rhto-GEM/releases/). All scripts are available from the ComplementaryData/Lopes2019 folder in the repository, and calculations were performed with RAVEN Toolbox (Wang et al. 2018) on MATLAB (The MathWorks Inc., Natick, MA, USA) using Gurobi solver (Gurobi Optimization Inc., Houston, TX, USA). Theoretical calculations were directly performed on unmodified rhto-GEM. Condition-specific models were reconstructed by incorporating experimental data from each bioreactor cultivation. The biomass equation was fitted to the measured total cellular protein and lipid content, while the storage carbohydrate component was scaled as required, and nucleotide levels were assumed to be unaffected.

To determine the flux distributions, carbon uptake, xylitol, and protein excretion were constraint to measured values, while the oxygen and carbon dioxide exchange fluxes were set as close to the measured oxygen transfer rate (OTR) and carbon dioxide transfer rate (CTR) as possible. Growth-associated maintenance was set to represent the polymerization cost of the biomass macromolecules (Förster et al. 2003). The specific growth rate was fixed to either the measured value or the highest achievable by simulation. ATP hydrolysis (i.e., non-growth-associated maintenance) was subsequently maximized as objective function. To estimate the internal flux distributions, the measured rates for the exchange reactions could vary by 5% (97.5–102.5%) followed by random sampling (*n* = 5000) of the solution space (Bordel et al. 2010). Detailed descriptions of all simulations are available in the Lopes2019 subfolder in the GitHub repository.

## Results

### C/N ratio studies under continuous cultivation mode

**Fig. 1:**
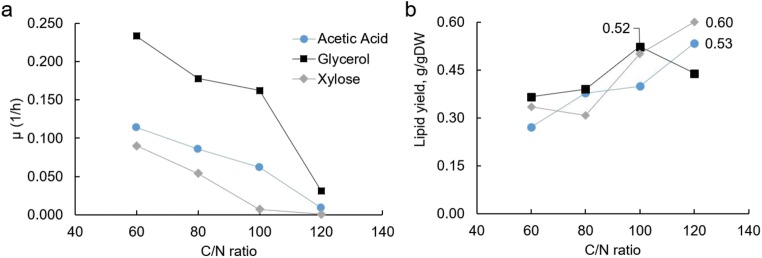
*R. toruloides* turbidostat cultivations under different C/N ratios and carbon sources (blue circles, acetic acid; black squares, glycerol; gray diamonds, xylose). **a** Specific growth rate (1/h); **b** lipid yield (g-lipid/gDW). Numeric values are highlighted for the highest lipid yields for every carbon source studied

To understand the effect of a C/N molar ratio on cell growth, turbidostat continuous cultivation mode was selected to avoid continually changing environmental conditions (including the C/N ratio). It allows one to achieve a steady physiological state at the maximal-specific growth rate (*μ*_max_) of the strain under the provided environmental conditions. The *μ*_max_ is strictly defined by the medium inflow rate required for the dilution of biomass concentration to a pre-set optical density value measured on-line. Four C/N ratios were selected for the study (60, 80, 100, and 120) under three different carbon sources—glycerol, acetic acid, and xylose. In all cultivations, an increase in the C/N ratio led to a decrease in the specific growth rate (Fig. 1a). At the same time, this behavior was accompanied with increased lipid yields (corresponding to lipid content), which reached up to 60% of the total cellular mass when grown on xylose at a C/N of 120 (Fig. 1b).

All data collected from turbidostat experiments were used as an input for flux balance analysis (FBA) based on a recent *R. toruloides* genome-scale model (GEM; Tiukova et al. 2019). FBA was carried out to (i) validate the feasibility of experimental results, (ii) identify potential additional products not measured with current analysis, (iii) understand the distribution of intracellular fluxes, (iv) determine the redox and energy constraints for the metabolism, and (v) calculate the maximal theoretical yield for lipid production for each of the three substrates studied. Our model showed that the highest theoretical lipid yields are 69, 61, and 50% of the consumed carbon directed into lipid synthesis for glycerol, xylose, and acetic acid, respectively. To simulate the internal flux distribution, the biomass composition and exchange fluxes were adjusted to the protein and lipid levels and measured rates from the bioreactors. The model could readily simulate the growth, carbon assimilation, and measured excretions, while some more variability could be observed in OTR and CTR values.

### Glycerol allows the highest growth and lipid production rates

**Fig. 2:**
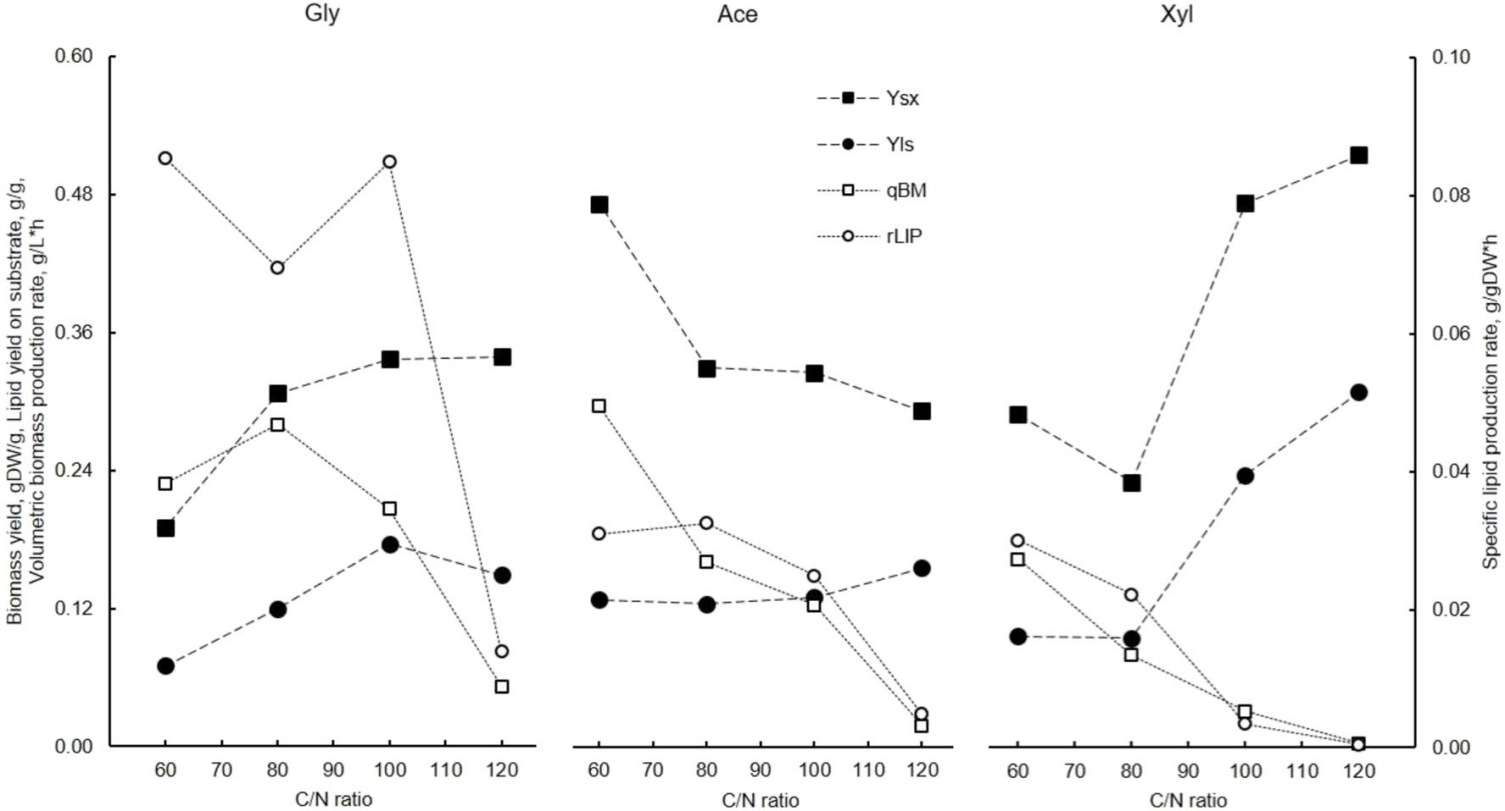
Effect of C/N ratio on yields and specific biomass and lipid production rates on the turbidostat cultivation of *R. toruloides* using different carbon sources. Biomass yield (*Y*_sx_), lipid yield on substrate (*Y*_ls_), and volumetric biomass (*q*_BM_) and specific lipid (*r*_LIP_) production rates are presented

**Fig. 3:**
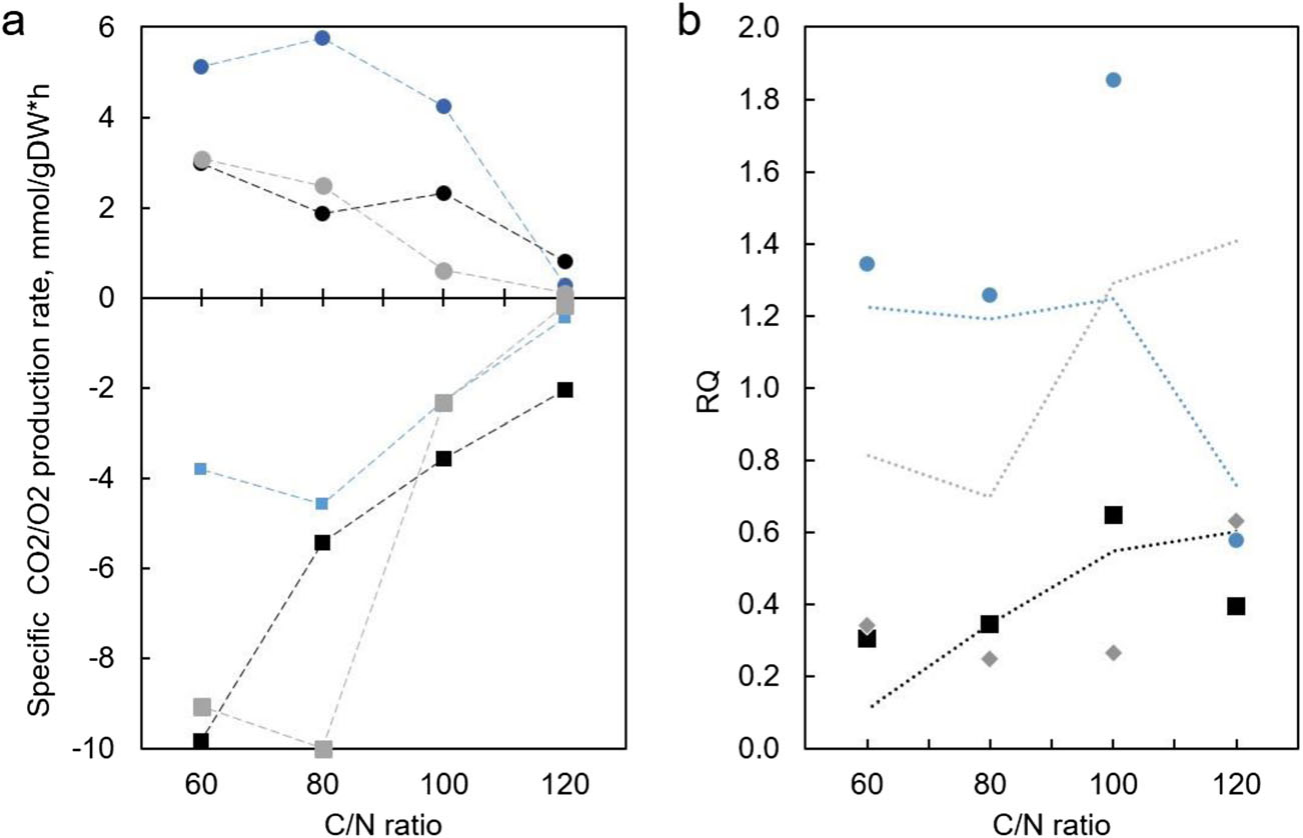
Specific CO_2_ (circles) and O_2_ (squares) production rates for acetic acid (blue), glycerol (black), and xylose (gray) growing *R. toruloides* strain under various C/N ratios (**a**). Note that negative production indicates consumption. RQ values for the same conditions (data points) and FBA predicted values (dotted lines; **b**)

Among the three carbon sources studied, glycerol allowed for the fastest growth on the basal mineral medium used in the experiments. Specific growth rates on glycerol reached 0.24 1/h at a C/N ratio of 60. In the case of glycerol, biomass yield increased with a decrease in the specific growth rate (i.e., increasing C/N ratios) from 0.19 to 0.34 gDW/g (Fig. 3). However, the cellular lipid content did not show the highest values compared with the other substrates studied due to the high specific growth rate on glycerol; the specific lipid production rates were the highest under all C/N ratios tested. The highest specific lipid production rates were observed under the C/N ratios between 60 and 100 (up to 0.085 g/gDCW*h), while the highest substrate to lipid yield was achieved at a C/N of 100 (Fig. 2). The specific lipid production rates under all C/N ratios studied were more than 2-fold higher compared with the analogous C/N ratios for acetic acid and xylose. Moreover, the highest specific oxygen consumption rates were observed for cells grown on glycerol (Fig. 3), although, the general correlation between oxygen consumption and lipid production rates over all conditions studied was insignificant (*R*^2^ = 0.31, *p* value = 0.06). Due to a sharp decrease in specific growth rate at C/N 120, all production and consumption rates decreased significantly. This emphasizes that strong N-limiting conditions are not likely to be optimal for industrial fed-batch production.

During growth on glycerol, carbon balance calculations based on biomass and CO_2_ production account for only 36–78% of the consumed glycerol. This indicates that there must be other excreted products in the environment that we were not able to identify with the HPLC-based carbohydrate analysis. We used the *R. toruloides* GEM to predict the potential byproducts of the cultivation process on glycerol. Random sampling of the solution space indicates that pyruvate, l-alanine, and oxaloacetate are potential byproducts when grown on glycerol (Supplementary Table S2). Interestingly, the measured RQ values, representing the ratio of CO_2_ produced to oxygen consumed by the cells, were unexpectedly low under these conditions, as the oxygen consumption rate exceeded CO_2_ production by 1.5- to 5-fold. Theoretically, an RQ of 0.9 would be expected in the case of the growth of *S. cerevisiae* on glycerol (Ochoa-Estopier et al. 2011), while we measured an RQ of 0.2–0.6 for *R. toruloides*. Feasibility of the measured RQ values was examined using GEM analysis. Constraining the model with the measured rates resulted in feasible simulations, where theoretical RQ values were detected in the same range as the measured results.

### Growth on acetic acid shows the highest biomass yield under lower C/N ratios

Growth on acetic acid resulted in the highest specific growth rate at C/N 60 of 0.11 1/h and the highest lipid content of 53% under the C/N 120. Contrary with studies with other carbon sources, biomass yield increased with an increase in specific growth rate and decrease in the C/N ratio (Fig. 2). The highest biomass yield (0.47 gDW/g), specific growth rate, and biomass production rate, with 0.30 gDW/(g-substrate*h) on acetic acid was detected at the lowest C/N ratio studied. The highest lipid content (and substrate to lipid ratio) was observed at C/N 120 and the highest specific lipid production rate at C/N 80 (Fig. 2).

Growth on acetic acid was unique in the dataset, as the CO_2_ production was the highest and oxygen consumption the lowest compared with the other conditions studied. This resulted in RQ values above 1. Because the degree of reduction for acetic acid is the same as glucose, RQ values close to 1 under fully respiratory conditions are expected. The carbon balance ranged between 80 and 115% under the C/N ratios studied and showed higher amounts of products (CO_2_) at C/N ratios between 60 and 100 (Supplementary Table S3).

To be able to close the carbon balance at C/N ratios 60–100, the model predicted CO_2_ production to be lower than the measured values, resulting in marginally lower predicted RQ values, which is in accordance with previous literature data (Fig. 3b). At a C/N ratio of 120, the model simulations predict that roughly 17% of the carbon taken up was excreted as glyoxylate, a metabolite not detectable with the analytical methods used in this study (Supplementary Tables S1 and S2).

### *R. toruloides* shows the highest lipid content in biomass when grown on xylose

Among the three carbon sources studied, *R. toruloides* displayed the highest lipid content in biomass on xylose at a C/N ratio of 120, 0.6 g/gDW (Fig. 1). Despite this, specific lipid production rates on xylose were the lowest compared with the other carbon sources, while the profile decreased with an increase in C/N. This was mainly a result of the relatively low biomass yields and specific growth rates under all the C/N ratios studied (Figs. 1 and 2, respectively). Because nitrogen was limiting growth, about a third of the xylose consumed was directly converted into byproducts—either xylitol or arabitol, which were indistinguishable with the analytical methods used. These were the only byproducts at the two higher C/N ratios studied, whereas based on carbon balance analysis, additional byproducts would be expected at the two lower C/N ratios.

FBA with xylose-consuming cells predicted that, in addition to xylitol production, a significant quantity of additional metabolites were produced under the C/N ratios 60 and 80 (Supplementary Table 1). While the model simulations suggest alanine and pyruvate as major overflow metabolites, it is also feasible that other metabolites could fulfill this role. However, no additional overflow metabolites were detected in the HPLC analysis in the xylose cultivations. The largest discrepancy between the measured and model-calculated oxygen consumption was determined when grown on xylose. Measured oxygen consumption was less than 2-fold higher compared with the predicted one at all tested C/N ratios, while the model was able to support the measured CO_2_ rates. High oxygen consumption would indicate higher glyoxylate flux to the tricarboxylic acid (TCA) cycle, which was not found to be feasible with the rhto-GEM.

Because the biomass collected from cultivations using xylose as a sole carbon source resulted in more intense visual coloration compared with the other substrates studied (Supplementary Fig. S1a), it was selected for the identification and quantification of carotenoids. Total carotenoid production seemed to increase with an increase in the C/N ratio (Supplementary Fig. S1b), reaching its highest concentration, 353.7 μg/gDW, at the C/N ratio of 120. Furthermore, the proportion of beta-carotene:torularhodin:torulene changed with C/N ratio, starting from 0.48:0.40:0.11 at a C/N ratio of 80 to 0.17:0.34:0.32 at the C/N ratio of 120 (Supplementary Fig. S1c).

### Modeling insights for intracellular flux patterns

Using the GEM of *R. toruloides* allowed us to look more precisely into the redox and energy metabolism of this oleaginous yeast. Because the experiments were carried out under nitrogen limitation conditions, we expected that the cells were not limited by the supply of energy. To validate this, we calculated the amount of ATP that the model could spent on non-growth-associated processes in each of the culture conditions, while the growth-associated energy requirements were near identical for all conditions (when normalized for growth rate). Overall, the models simulate higher ATP turnover on xylose and glycerol in respect to acetic acid grown cells (Supplementary Table S2). This suggests that cells growing on acetic acid have a more efficient metabolism and physiology, operating at near-maximum efficient levels in terms of biomass production.

**Fig. 4:**
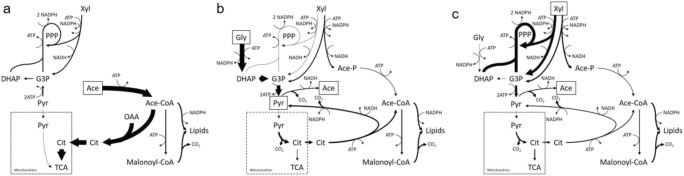
Condition-dependent flux predictions in *R. toruloides* turbidostat cultivations on different carbon sources: **a** glycerol, **b** acetic acid, and **c** xylose. Fluxes are calculated using flux balance analysis on *R. toruloides* genome-scale model. Arrow width represents proportional flux values relative to carbon uptake flux. Precise values and reaction names can be found from Supplementary Table S6. Gly, glycerol; Xyl, xylose; Ace, acetic acid; PPP, pentose phosphate pathway; DHAP, dihydroxyacetone phosphate; Pyr, pyruvate; Cit, citrate; TCA, tricarboxylic acid cycle; Ace-CoA, acetil-CoA; Ace-P, acetyl phosphate; G3P, glycerol 3-phosphate; OAA, oxaloacetate

Fatty acid biosynthesis is often limited by the supply of NADPH, as the elongation of fatty acids during their biosynthesis by two carbon units requires two NADPH molecules. Genome-scale metabolic modeling allowed us to investigate the production pathways of NADPH in *R. toruloides*, but only limited variation in the distribution of NADPH producing and consuming reactions could be observed while growing on the same substrate but under different C/N ratios. The most significant difference was detected when grown on glycerol, where clearly higher NADPH consumption was predicted in protein biosynthesis under the lowest C/N ratio studied (Supplementary Table S4). At the same time, significant differences in NADPH production were found to be carbon source dependent. Glycerol-grown cells produce the majority of NADPH via NADPH-dependent glycerol dehydrogenase, while acetate-grown cells mainly use TCA-cycle related NADPH production pathways, i.e., NADPH-dependent isocitrate dehydrogenase, malic enzyme, and NADPH-dependent succinate-semialdehyde dehydrogenase (Fig. 4, summarized reactions on Supplementary Table S5 and all reactions are shown on Supplementary Table S6). Xylose-grown cells mainly utilize the oxidative part of the pentose phosphate pathway: glucose 6-phosphate dehydrogenase and phosphogluconate dehydrogenase.

Phosphoketolase catalyzes redox-independent cleavage of sugar phosphates, including the conversion of d-xylulose 5-phosphate into glyceraldehyde 3-phosphate and acetyl-phosphate and is a more efficient way to provide acetyl-CoA for fatty acid biosynthesis. The latter enzyme is known in many bacteria and eukarya, and its presence has been demonstrated also in *R. toruloides* (Evans and Rafledge 1984; Sánchez et al. 2010). While model simulations estimated that phosphoketolase activity could vary during growth on xylose, on average 20–32% of the consumed xylose is metabolized via the phosphoketolase pathway depending on the C/N ratio, while the remaining xylose uses the traditional pentose phosphate pathway fluxes for this conversion. Phosphoketolase is potentially also active when grown on glycerol, where, on average, 20% of the consumed carbon was estimated to use this enzyme. ATP/citrate lyase (ACL), an enzyme characteristic of oleaginous yeasts, breaks cytosolic citrate into oxaloacetate and acetyl-CoA, representing an alternative method of producing acetyl-CoA. We determined that under a high C/N ratio on xylose, up to 32% of the consumed carbon is metabolized via ACL, and up to 38% on glycerol-grown cells. Due to the direct conversion of acetic acid into acetyl-CoA via acetyl-CoA synthase, ACL is used minimally (Supplementary Table S6). Also, the malic enzyme, which converts malate into pyruvate while producing NADPH, has been speculated to play a crucial role in lipid synthesis in oleaginous yeast. In our model, malic enzyme converts up to 3.5% of the consumed carbon flux on acetate-grown cells (corresponds up to 5.5% of the total NADPH production) but was almost not used under glycerol and xylose conditions.

### Comparison of lipid production in batch and fed-batch conditions using glycerol as a sole carbon source

Based on turbidostat experiments with different C/N ratios and the results from metabolic modeling, glycerol was identified as the carbon source that produces lipids at the highest rate. Based on this, we went on to conduct batch and fed-batch experiments to measure lipid content and production rates using a more industrially relevant approach. Batch experiments in shake-flasks using 70 g/L glycerol revealed maximal biomass and lipid content after 120 h of cultivation providing 17.7 g/L of biomass and a lipid content of 42.13% (in terms of lipid yield *Y*_l/*x*_ = 0.421 g/gDW), respectively. Regarding productivity values, maximal results were observed within 48 h of cultivation with *r*_*x*_ of 0.0208 g/(gDW*h) and *r*_lip_ and *r*_gly_ of 0.00487 and 0.0310 g/(gDW*h), respectively.

In preparation for fed-batch cultivation with an exponential feeding strategy (constant *μ*), information from turbidostat experiments was used to select the most suitable C/N ratio for the feed medium. Because C/N ratios between 60 and 100 showed lipid production rates within the same range, we selected a C/N ratio of 60 to achieve the highest lipid content under fed-batch conditions.

Lipid production was evaluated under two different C/N ratios during batch and two different growth rates in the fed-batch mode, 0.03 and 0.05 1/h. The batch-phase with a C/N ratio of 60 (mol/mol) resulted in *μ*_max_ of 0.048 ± 0.005 1/h, 14.6 ± 0.5 gDW/L of biomass, 77% of glycerol uptake, lipid titer, and lipid content of 6.0 ± 1.4 g/L and 41.3% ± 9.9, respectively. The glycerol was not completely exhausted, probably due to nitrogen limitation. The averaged volumetric productivity of lipids was 0.049 ± 0.013 g/L*h, while the specific productivity was 0.003 ± 0.001 g lipids/gDW*h. The yield of substrate converted to lipids was 0.18 g lipids/g glycerol and the glycerol consumption per biomass was 2.3 g glycerol/g DW. A lower C/N ratio (17 mol/mol) during batch resulted in complete glycerol uptake, similar *μ*_max_ (0.044 ± 0.005 1/h), but higher glycerol consumption per biomass formation yield (3 g glycerol/gDW) and lower final biomass (10.9 ± 0.1 gDW/L).

**Fig. 5:**
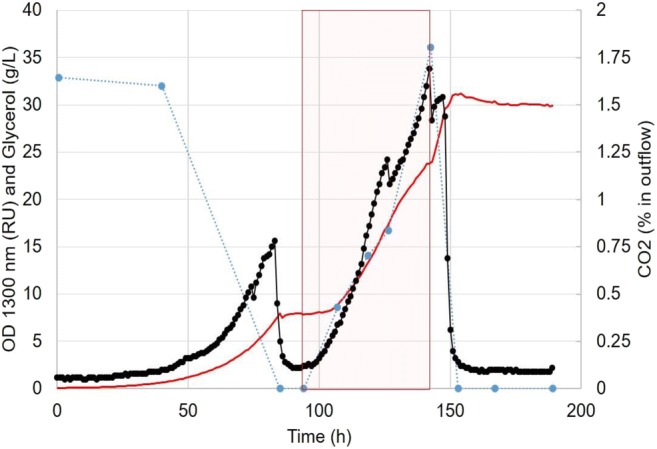
*R. toruloides* fed-batch fermentation on glycerol, where CO_2_ (black line with filled circles), OD (red line), and glycerol concentration in the reactor (open blue circles) are pictured. Red rectangle indicates the period when glycerol feeding with a constant specific growth rate of 0.05 1/h was applied (experimental set-up no. 3, “Materials and methods”)

For the experiments with an initial batch phase carried out at C/N 60 mol/mol, the fed-batch phase for the cultivation using a *μ*_0_ of 0.03 1/h (Supplementary Fig. S2) for the feeding algorithm was carried out for 53.5 h. When using a *μ*_0_ of 0.05 1/h the fed-batch phase was carried out for both 29 h (Supplementary Fig. S3, initial C/N 60 mol/mol) and 48 h (Fig. 5, initial C/N of 17 mol/mol). On all experimental set-ups, biomass increased around 2.4-fold, the lipid content of the cells remained around 36–39% and glycerol accumulated during the feeding stage reaching around 35–50 g/L. During the feeding stage (Fig. 5), we were able to see four distinct growth phases. First, when feeding was initiated, cells rested for approximately 10 h and no significant growth was observed. During the second and third growth phases, cells were grown with a specific growth rate of 0.035 and 0.02 1/h, respectively. And during the fourth growth phase when more than 30 g/L of glycerol had accumulated in the environment, the specific growth rate of the cells increased to the same value observed during the batch phase. The specific lipid productivity at this stage was on average 0.010 g lipid/gDW*h. The maximum lipid titer was obtained after the end of the fed-batch 17.1 ± 1.2 g/L (content of 36.7% ± 3.3) and 20.0 ± 0.8 g/L (content of 42.9% ± 1.8) for *μ*_0_ of 0.05 and 0.03 1/h, respectively.

## Discussion

Nitrogen limitation conditions are commonly used to induce higher accumulation of lipids in various oleaginous organisms (Papanikolaou and Aggelis 2011). Previously, it has been demonstrated how different C/N ratios influence lipid accumulation under dynamic batch conditions.

The crucial finding that higher C/N ratios resulted in higher lipid content, but lower specific growth rate has also been described previously for other oleaginous yeasts (Papanikolaou and Aggelis 2002; Shen et al. 2013; Béligon et al. 2016). When aiming at the feasibility of producing oleochemicals from single cell oil using low-cost substrates, productivity and specific production rates must be strongly considered. Concerning lipid productivity, the best production rates were observed with glycerol as a sole substrate at a C/N ratio of 100, resulting in *r*_lip_ of 0.085 g/gDCW*h. The specific lipid production rate observed in this work is not only higher than other studies focused on continuous lipid production from glycerol (*r*_lip_ of 0.018 g/gDW*h was observed by Papanikolaou and Aggelis 2002) but also one of the highest reported in a continuous process (*r*_lip_ of 0.021 g/gDW*h was observed by Béligon et al. (2016) using the same acetic acid as substrate at a C/N ratio of 50 and the yeast *Cryptococcus curvatus*).

Fed-batch processes allow one to achieve high cell densities and high titers of products, which are crucial for microbial oil production because the production rate and titer play an important role in its final price (Meesters et al. 1996). There are a few studies that have explored fed-batch cultivation using glycerol as a substrate for *R. toruloides* (Li et al. 2007; Uçkun Kiran et al. 2013; Dias et al. 2015; Leiva-Candia et al. 2015; Signori et al. 2016), yet none of these applied a constant *μ* in their feeding strategy. Also, all these studies and the ones mentioned previously used either yeast extract or another rich nitrogen source for cell proliferation. Although we used a relatively poor nitrogen source in the current study—ammonium sulfate—we were able to reach a higher final biomass and lipid titers than the ones obtained by Uçkun Kiran et al. (2013) and Leiva-Candia et al. (2015), with 31.1 and 37.4 gDW/L and 12.9 and 19.2 g/L of lipids, respectively. Although fed-batch allows one to achieve higher biomass and titers than in continuous cultivation mode, the production rates and lipid yield were smaller. Low lipid content during fed batch was also observed by Meesters et al. (1996) and Uçkun Kiran et al. (2013). According to the latter, this was an indication that cells utilized more glycerol to grow faster rather than accumulating higher levels of lipids. Li et al. (2007) reported a fed-batch strategy for microbial oil production with *R. toruloides* that used a nutrient-rich media for cell proliferation during batch followed by feeding only pure glucose. These authors measured the concentration of inorganic nitrogen in the medium over time and found a correlation between the course of ammonium depletion with the onset of lipid accumulation. Exhaustion of nitrogen greatly promoted lipid accumulation from 18.1 to 43.8% (*w*/*w*). When comparing data between continuous and fed-batch cultivation using glycerol as a carbon source, lipid content was similar on both modes, but the continuous mode was superior in terms of specific and volumetric lipid production rates, while fed batch resulted in higher titers.

Maximal theoretical yields calculated for different carbon sources indicate that glycerol provides the highest lipid production, where 69% of consumed carbon could theoretically be directed towards lipid synthesis. Although *R. toruloides* does not secrete lipids, in silico calculations of theoretically maximal lipid conversion assumes an efficient lipid transport to the extracellular environment and minimal growth, i.e., wasting carbon for biomass production. Therefore, we have currently achieved only about 25% of the theoretical maximum yield, which indicates there is sufficient room for process improvement and this approach still holds promise to become economically feasible. The highest carbon to lipid conversion rate in our studies was achieved on xylose, where approximately a third of the maximal theoretical yield was presented.

A techno-economic analysis on microbial oil production has been carried out by Koutinas et al. (2014) where after accounting for the production, separation, lipid purification, and capital and employment costs, they estimated a price of a $5.5/kg oil. Although, this price is two to four times more expensive when compared with plant oils, these prices were calculated based on the reported productivity (Koutinas et al. 2014) and glucose price of $400/t. In the current study, we have already shown higher productivity compared with Koutinas et al. (2014) and have focused on the utilization of less preferred and cheaper carbon sources as xylose and acetic acid from the hemicellulose hydrolysate and glycerol as the byproduct of biodiesel. Taking into account the theoretical maximal yield of lipid production with *R. toruloides* calculated using the genome-scale models, a significant increase in lipid production could be expected, especially when lipid excretion could be effectively established. Ledesma-Amaro et al. (2016) showed that secreting fatty acids in metabolic engineered *Yarrowia lipolytica* not only allowed going beyond the maximum lipid accumulation capacity (as it is an intracellular product), but also facilitated its recovery, which might represent 40–80% of the total production cost.

According to Koutinas et al. (2014), the co-production of high-value compounds together with oleochemicals is imperative when considering the economic viability of biodiesel production from oleaginous yeasts. In this sense, *R. toruloides* offers a natural way to produce carotenoids (Marova et al. 2012), which are pigments that feature an important biological function as precursors for vitamin A (β-carotene), demonstrate potential anti-cancer properties, and can stimulate the immune system (Yamada et al. 2018). Carotenoids are commonly extracted from vegetables or produced chemically from petroleum-derivatives, however, both methods present drawbacks, such as being unable to fill the world demand or unfriendly to the environment, respectively (Marova et al. 2012). Production of carotenoids by *R. toruloides* can be a potential option to overcome both drawbacks and improve the economic viability of oleochemical production with microorganisms.

Even without focusing on carotenoid production, the results obtained in the present work using xylose and C/N of 120 (mol/mol) was comparable with some studies that aimed at carotenoid production. Dias et al. (2015) achieved a total carotenoid concentration of 0.29 mg/gDW during fed-batch cultivation of *R. toruloides* NCYC 921, result slightly lower than the maximum observed here (0.35 mg/gDW). Zhang et al. (2014) observed the production of 0.048 mg/g during cultivation of *Rhodotorula glutinis* in a photobioreactor, and even with oxidative stress this result was almost seven times lower than that obtained in this present work. Martins et al. (2018) obtained 0.420 mg/gDW of carotenoid during *R. toruloides* fed-batch cultivation using carob pulp syrup as a carbon source. Although carotenoid production reported by those authors was almost 1.2 times higher than the production reported in this work, they used higher concentrations of substrate, carob syrup at 548.7 g/L, compared with 10 g/L of xylose.

Regarding xylose cultivation, the favored torularhodin and torulene production at higher C/N might surpass the low *r*_lip_ and *r*_x_ values obtained, by offering product diversification and possible profitability as these non-conventional carotenoids are not produced at a large scale and only a number of yeasts and fungi can synthesize them naturally (Kot et al. 2018). Furthermore, studies show that they possess interesting properties against cancer (Du et al. 2016), microbial activity (Ungureanu et al. 2016), and as antioxidants (Dimitrova et al. 2013). In terms of processes, results obtained with *R. toruloides* show a possibility to control the profile of carotenoids during cultivation throughout the change on C/N ratio and dilution rate.

The data presented in this work can be used for the techno-economic analysis of microbial oil production from low-cost substrates. Glycerol as a substrate was superior in terms of specific lipid production rates, but xylose offered product diversification (carotenoids) and higher lipid accumulation. The genome scale model predicted that lipid production using the substrates studied can be vastly improved by both process and metabolic engineering, especially for glycerol. GEM analysis also provided possible targets for the metabolic engineering, such as improving the utilization of the malic enzyme (increasing NADPH supply) and the phosphoketolase pathway (carbon saving).

## Electronic supplementary material

ESM 1 (PDF 584 kb)

ESM 2 (XLSX 697 kb)
