# Supplementary material for: C/N ratio and carbon source-dependent lipid production profiling in Rhodotorula toruloides
Source: Appl Microbiol Biotechnol. 2020 Jan 24;104(6):2639–49. doi: 10.1007/s00253-020-10386-5 (PMC7044259; doi:10.1007/s00253-020-10386-5)
Supplement: Supplementary file 1 — (PDF 584 kb) [file 253_2020_10386_MOESM1_ESM.pdf]

Applied Microbiology and Biotechnology

Supplementary Figures

C/N ratio and carbon source-dependent lipid production profiling in *Rhodotorula toruloides*

Helberth Júnnior Santos Lopes[1,2,*], Nemailla Bonturi[1,*], Eduard Johannes Kerkhoven[3], Everson Alves Miranda[2], Petri-Jaan Lahtvee[1]

[1]Institute of Technology, University of Tartu, Tartu, Estonia

[2] Department of Materials and Bioprocess Engineering, School of Chemical Engineering, State University of Campinas, Brazil,

[3] Department of Biology and Biological Engineering, Chalmers University of Technology, Göteborg, Sweden

[*]Contributed equally

Emails: helberthlopes@hotmail.com, bonturi@ut.ee, eduardk@chalmers.se, everson@unicamp.br, lahtvee@ut.ee

ORCID

HJSL 0000-0003-2864-4038

NB 0000-0003-4867-8286

EJK 0000-0002-3593-5792

EAM 0000-0002-8594-2130

PJL 0000-0002-3327-3190

Corresponding author:

Petri-Jaan Lahtvee

Email: lahtvee@ut.ee

Tel: +372 5088 117

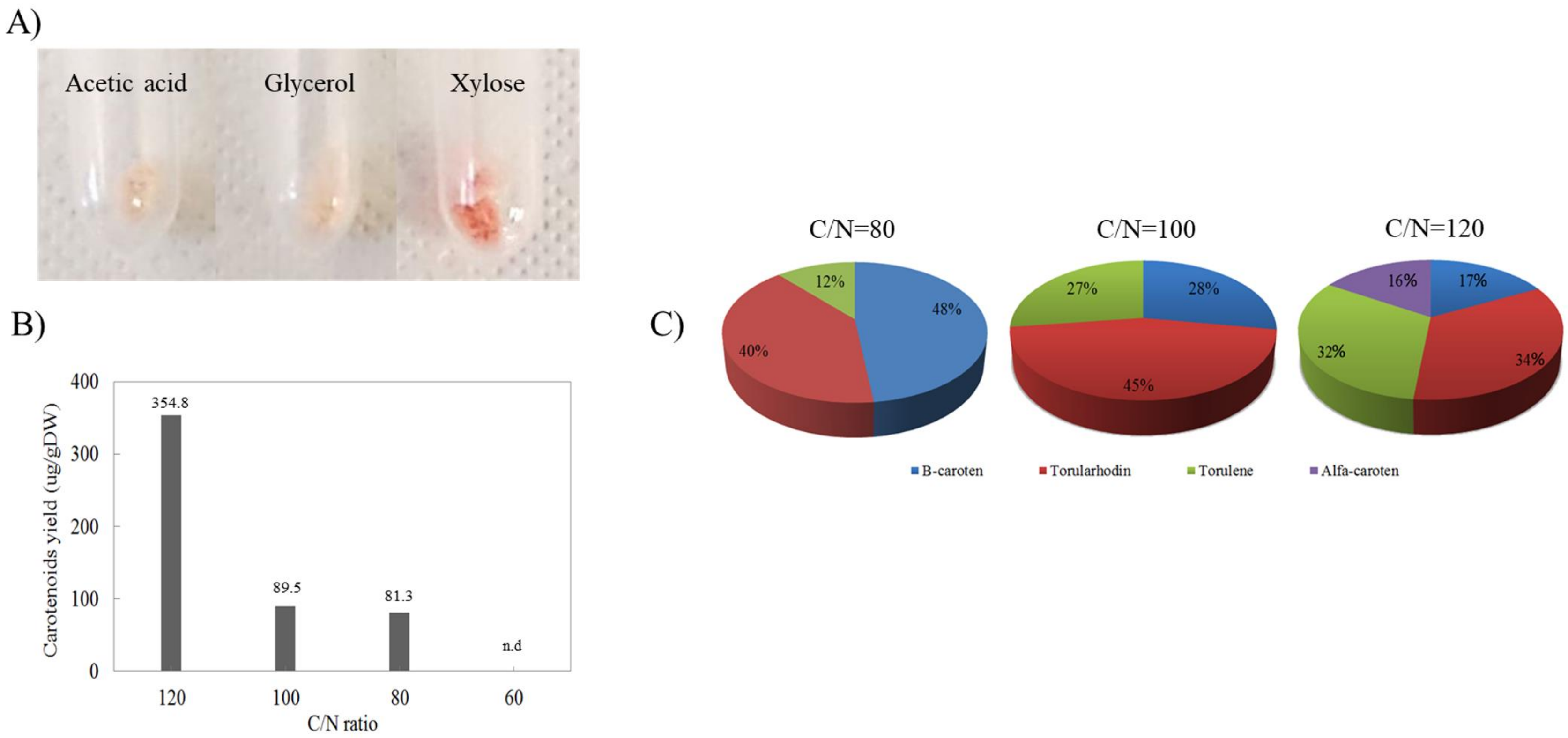

Fig S1. A) Biomass cell pellets from different the turbidostat cultivation with different substrates at C/N of 100 (mol/mol). **B)** Total caretonoids concentration (ug/gDW) and **C)** composition (%) from the turbidostat cultivation using xylose as carbon source at different C/N ratios.

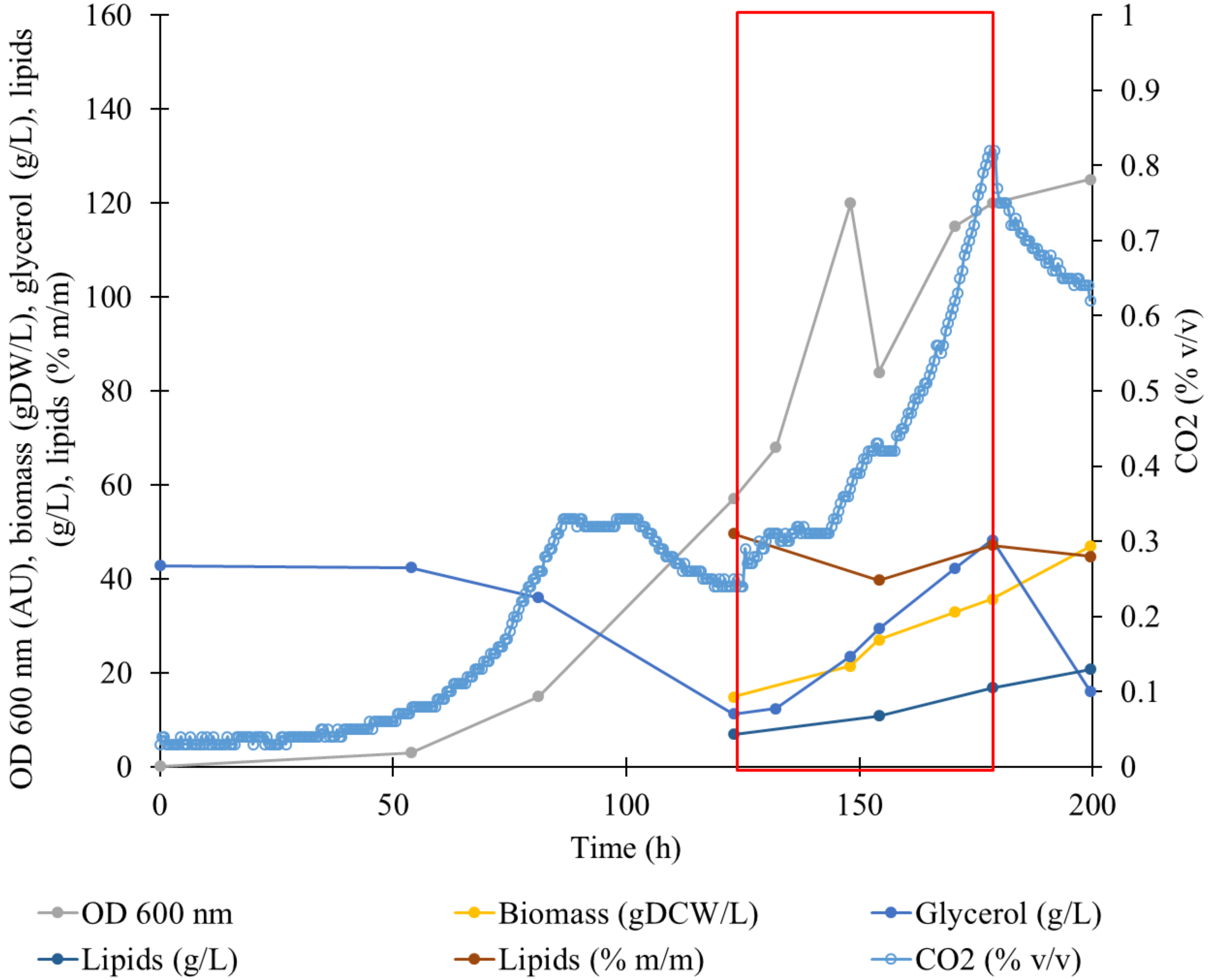

Fig S2. Cultivation profile of the fed-batch cultivation using glycerol as carbon source and μ_0 of 0.03 1/h

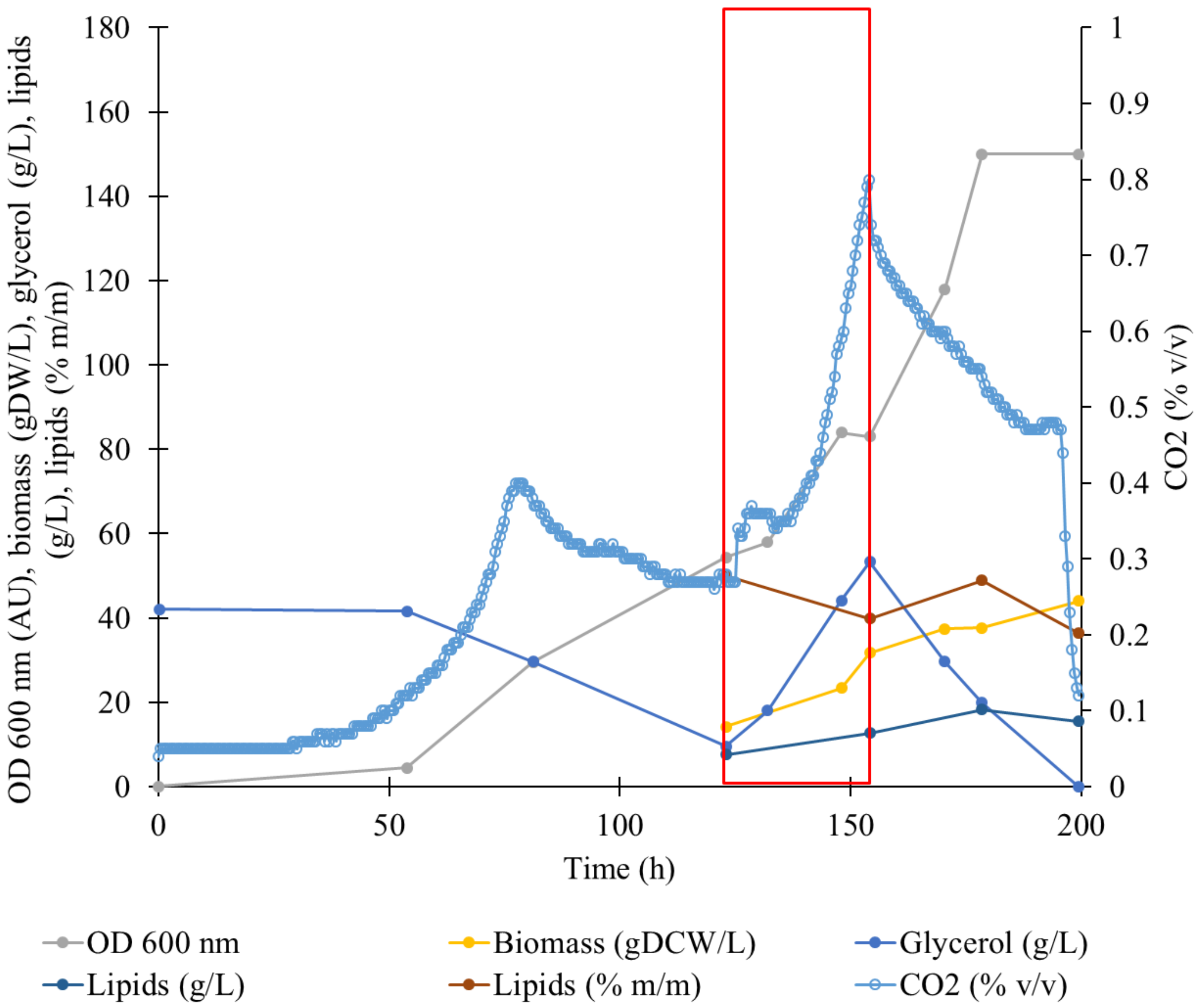

Fig S3. Cultivation profile of the fed-batch cultivation using glycerol as carbon source and μ_0 of 0.05 1/h
